# *In Vivo* Nitric Oxide Synthase Inhibitors Can Be Deprived of This Activity: Unexpected Influence of the Tetrachloroplatinate(II) Counteranion. Crystal Structures of Bis(S-Methyl-Isothiouronium)-N,N′-Bis(3-Guanidinopropyl)Piperazinium and Hexamidinium Tetrachloroplatinates(II) Salts

**DOI:** 10.1155/MBD.1998.127

**Published:** 1998

**Authors:** Georges Morgant, Bernard Viossat, Monique Roch-Arveiller, Patrice Prognon, Jean-Paul Giroud, Jean-Charles Lancelot, Max Robba, Dung Nguyen Huy

**Affiliations:** 1 Laboratoire de Cristallochimie Bioinorganique Faculté de Pharmacie Paris XI, 5 rue-Baptiste Clément Châtenay-Malabry Paris F-92290 France; 2 Laboratoire de Biochimie Hôpital Armand Trousseau Paris France; 3 Laboratoire de Chimie Générale Faculté de Médecine et de Pharmacie Poitiers France; 4 CNRS URA 1534 Laboratoire de Pharmacologie Hôpital Cochin Paris France; 5 Laboratoire de Chimie Analgtique Faculté de Pharmacie Paris Xl Châtenay-Malabry Paris France; 6 Laboratoire de Chimie Thérapeutique Faculté de Pharmacie Caen France

## Abstract

The synthesis and crystal structures of bis(S-methylisothiouronium) (MSTUH)^+^, N,N′-bis((3- guanidinopropyl)piperazinium (PipeC3GuaH4)^4+^ and hexamidinium (HexaH2)^2+^ tetrachloro platinate(ll) salts ( called hereafter PtMSTU, PtPipeC3Gua and PtHexa respectively ) were investigated. These compounds contain the “amidine” function ( - C(=NH)NH_2_ ) in which the H atoms supplied by the acid have become attached to the imino group of each terminal amidino function. Moreover, in PtPipeC3Gua, the nitrogen atoms of the chair-piperazine moiety are also protonated. The influence of tetrachloroplatinate(ll) counteranion ( versus sulfate, nitrate and diisethionate ) in the *in vivo* nitrite inhibition by the (MSTUH)^+^, (PipeC3GuaH4)^4+^ and (HexaH2)^2+^ cations was investigated. The three tetrachloroplatinate(ll) salts, unexpectedly, do not inhibit significantly the *in vivo* nitrite production in comparison with the other salts (sulfate, nitrate and diisethionate and their corresponding previous countercations) which exhibit NO synthase inhibition, especially bis(S-methylisothiouronium) sulfate, a selective and potent inducible NO synthase (iNOS) inhibitor commonly used as standard.


### *IN VIVO* NITRIC OXIDE SYNTHASE INHIBITORS CAN BE DEPRIVED OF THIS ACTIVITY: UNEXPECTED INFLUENCE OF THE TETRACHLOROPLATINATE(II) COUNTERANION. CRYSTAL STRUCTURES OF BIS(S-METHYL-ISOTHIOURONIUM)-N,N'-BIS(3-GUANIDINOPROPYL)PIPERAZINIUM AND HEXAMIDINIUM TETRACHLOROPLATINATES(II) SALTS

Georges Morgant[1,2], Bernard Viossat[3], Monique Roch-Arveiller[4], Patrice Prognon[5], Jean-Paul Giroud[4], Jean-Charles Lancelot[6], Max Robba[6] and Dung Nguyen Huy*[1]

[1] Laboratoire de Cristallochimie Bioinorganique, Faculté de Pharmacie Paris XI, 5, rue-Baptiste Clément, F-92290 Châtenay-Malabry, France. E-mail: Morgant@cep.u-psud.fr

[2] Laboratoire de Biochimie, Hôpital Armand Trousseau, Paris, France

[3] Laboratoire de Chimie Générale, Faculté de Médecine et de Pharmacie, Poitiers, France

[4] CNRS URA 1534, Laboratoire de Pharmacologie, Hôpital Cochin, Paris, France

[5] Laboratoire de Chimie Analytique, Faculté de Pharmacie Paris XI, Châtenay-Malabry, France

[6] Laboratoire de Chimie Thérapeutique, Faculté de Pharmacie, Caen, France

**Abstract**

The synthesis and crystal structures of bis(S-methylisothiouronium) $(MSTUH)^+$, N,N'-bis((3-guanidinopropyl)piperazinium $(PipeC3GuaH4)^{4+}$ and hexamidinium $(HexaH2)^{2+}$ tetrachloro platinate(II) salts ( called hereafter PtMSTU, PtPipeC3Gua and PtHexa respectively ) were investigated. These compounds contain the "amidine" function ( - $C(=NH)NH_2$ ) in which the H atoms supplied by the acid have become attached to the imino group of each terminal amidino function. Moreover, in PtPipeC3Gua, the nitrogen atoms of the chair-piperazine moiety are also protonated. The influence of tetrachloroplatinate(II) counteranion ( versus sulfate, nitrate and diisethionate ) in the *in vivo* nitrite inhibition by the $(MSTUH)^+$, $(PipeC3GuaH4)^{4+}$ and $(HexaH2)^{2+}$ cations was investigated. The three tetrachloroplatinate(II) salts, unexpectedly, do not inhibit significantly the *in vivo* nitrite production in comparison with the other salts ( sulfate, nitrate and diisethionate and their corresponding previous countercations ) which exhibit NO synthase inhibition, especially bis(S-methylisothiouronium) sulfate, a selective and potent inducible NO synthase ( iNOS ) inhibitor commonly used as standard.

### 1. Introduction

The free radical and paramagnetic molecule nitric oxide (NO) is produced enzymatically in physiological and pathological conditions by a family of NO synthases ( NOS ) which catalyze the oxidation of L-arginine ( from the guanidino group ) to L-citrulline. These enzymes include constitutive ( cNOS ) and inducible ( iNOS ) forms. NO from endothelial constitutive isoform ( ecNOS ) is a potent vasodilator and was identified with the endothelium dependent relaxing factor ( EDRF ) which mediates vascular relaxation in response to acetylcholine and substance P [ 1 ]. NO is also implicated in the regulation of blood pressure and inhibits platelet aggregation and leukocyte adhesion [ 2 ]. Endothelial overproduction of NO is implicated in atherosclerosis, diabetes and hypertension [ 3 ]. The continuous release of NO from the ecNOS keeps the vasculature in a continuous state of active vasodilatation.

The neuronal enzyme is found in brain and in peripheral nervous cells. In addition to its role in blood vessels, NO is a neurotransmitter found in high density in emotion-regulating brain regions [ 4 ]. NO from the constitutive neuronal isoform is implicated in neurodegeneration and neuroinjury. The inducible form iNOS is expressed in response to immunological stimuli in macrophages and other cells ( smooth muscle cells, hepatocytes ) and produces large amounts of NO which can act as cytotoxic molecule [ 5 ]. The cytotoxicity of NO from activated macrophages plays a role in their antimicrobial activity. NO synthesized by lymphocytes is greatly implicated in apoptosis phenomena. Recent studies showed that treatment with exogenous NO produces DNA strand breaks [ 6 ]. The generation of endogenous NO in large amount is implicated in circulatory shock secondary to the release of the proinflammatory cytokines ( tumor necrosis factor TNF$\alpha$ and interleukine 1 IL1 ) [ 7 ]. Antiinflammatory cytokines including IL4, IL10 and IL13 inhibit the induction of iNOS. Overproduction of NO by iNOS has been implicated in the chronic inflammation, vascular dysfunction in diabetes and transplant rejection. Novel therapeutic approaches could be designed to modulate the NO biosynthesis : drugs with high selectivity for iNOS inhibition may be useful for treatment of neurodegenerative disorders, chronic inflammatory diseases and septic shock whereas drugs inhibiting brain NO synthase ( bNOS ) may be protective against neuroinjury.

Many types of NOS inhibitors are known. The generation of NO can be inhibited by analogues of the substrate L-arginine ; N$\omega$-propyl-L-arginine was recently described as a potent and neuronal nitric oxide synthase inhibitor [ 8 ]. Other classes of compounds that are not amino acids but containing the " amidine " function ( - C = (NH) $NH_2$ ), such as amidines [ 9 ], S-alkylisothioureas [ 10 ] and substituted aminoguanidines and aminoisothioureas [ 11 ], are reported to be inhibitors of NOS. Bis(S-methylisothiouronium) sulfate is known to be a potent and preferential inhibitor of human and rat iNOS ; it exerts beneficial effects and improves survival in a rodent model of septic shock [ 12 ]. More recently, 1H-pyrazole-1-carboxamidine hydrochloride ( or PCA ) and its substituted derivatives have been shown to inhibit NOS with an increased selectivity toward iNOS when the pyrazole ring is substituted, as in 4-methyl-PCA [ 13 ].

In the course of our investigations, we have previously examined the ability of some N,N'-(alkane-1,$\omega$-diyl)bis(guanidinium) and N,N'-bis(3-guanidinopropylpiperazine) dinitrates to inhibit nitrite production in rat pleural cavity [ 14 ]. Moreover, in a previous paper, we have demonstrated that N,N'-(octane-1,8-diyl)bis(guanidinium) tetrachloroplatinate(II) exhibits cytostatic effects against trophozoites of an *Acanthamoeba* strain, in contrast to the tetrachloropalladate(II) salt which lacks this effect [ 15 ].

This prompted us to study the possible modifications of pharmacological properties of NOS inhibitors when pharmacologically inert anions ( sulfate, nitrate, chloride ) were replaced by other counteranions such as tetrachloroplatinate(II) which might reverse or enhance the expected properties.

Hexamidine diisethionate is a drug well-known for its antimicrobial effects which result from cationic surface active properties as a consequence of the bipolar structure of its cation. To our knowledge, its NOS inhibitor properties have not been described.

The aim of this work was to compare the anti-NO synthase properties of bis(S-methylisothiouronium) sulfate, ( a compound chosen as a reference NO inhibiting drug in our previous experiments ), N,N'-bis(3-guanidinopropylpiperazine) dinitrate and hexamidine diisethionate versus the corresponding tetrachloroplatinate(II) salts, called hereafter PtMSTU, PtPipeC3Gua and PtHexa respectively. All these compounds contain the amidine function.

### 2. Results and discussion.

### 2.1. Descriptions of the structures.

In the three tetrachloroplatinate(II) salts, the " amidine " function ( - C(=NH)NH2 ) was protonated with the H atoms supplied by the acid at the imino group of each terminal amidino function. ( Tables 1 and 2 )

2.1.1. *Description of the PtMSTU structure.* ( Figure 1a )

The asymmetric unit consists of half of a square planar $[PtCl_4]^{2-}$ anion and one $[MSTUH]^+$ cation. The S(1) - C(2) bond length of 1.782(7) Å is in good agreement with the length of a pure S - C single bond ( 1.82 Å ) while the S(1) - C(1) bond length ( 1.730(6) Å ) is significantly shorter and indicates an approximately 50% double-bond character. The atoms S(1), C(1), N(1) and N(2) of the isothiouronium cation were coplanar and the methyl carbon C(2) is 0.17 Å out of this plane, to which the S(1) - C(2) bond is inclined at an angle of 5.6°. Between the two N - C - S bond angles, there is a difference of 7.6°. The larger angle is the one to which the remote substituent on the sulfur atom stands in a *syn*-relationship as shown by the torsion angles of 6.02° for the N(2) - C(1) - S(1) - C(2) and of -174.48° for the N(1) - C(1) - S(1) - C(2). Similar observations have been made on the crystal structures of bis(S-methylisothiouronium) sulfate [ 16 ] and S-methylthiouronium p-chlorobenzoate [ 17 ].

All the amidinium H atoms are involved in hydrogen bonding to $Cl(1^{i})$, $Cl(1^{iii})$, $Cl(2^{i})$ and $Cl(2^{ii})$. The most noticeable feature is that there is one bifurcated hydrogen bond involving H(102) ( attached to N(1) ) with $Cl(1^{i})$ and $Cl(2^{ii})$ ( symmetry code i : - x, 1 - y, 1 - z ; ii : 1 - x, 1 - y, 1 - z ; iii : x, 1 + y, z ).

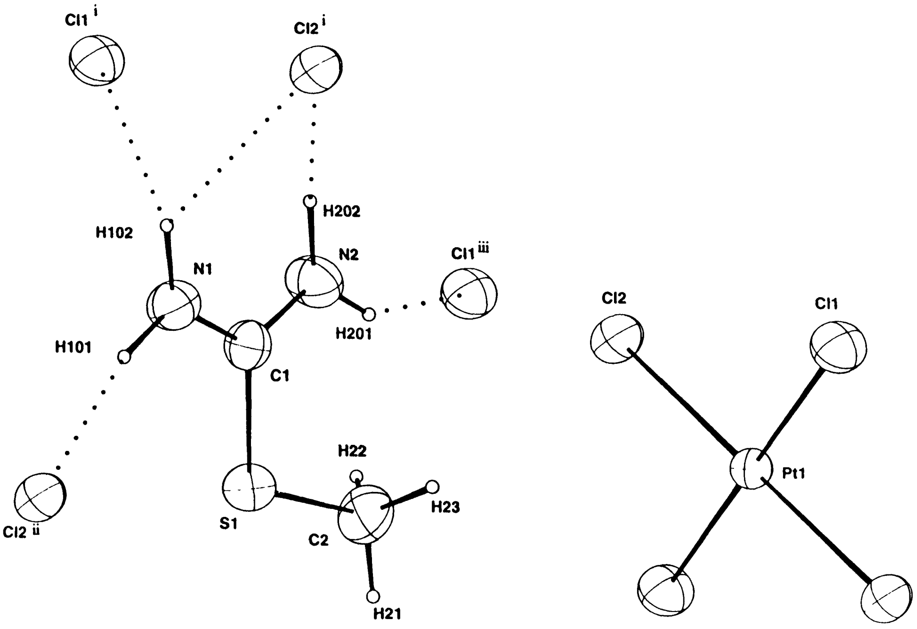

Figure 1a : Molecular structure of bis(S-methylisothiouronium) tetrachloroplatinate(II), showing the atom numbering scheme.

2.1.2. *Description of the PtPipeC3Gua structure.* ( Figure 1b )

The asymmetric unit consists of two half crystallographically independent and square planar $[PtCl_4]^{2-}$ anions and one half $[PipeC3GuaH4]^{4+}$ cation where PipeC3GuaH4 is the tetraprotonated form of the organic ligand N,N'-bis(3-guanidinopropyl)piperazine. The crystal structure shows that i) the organic cation presents a center of symmetry. ii) the four H atoms supplied by the acid have become attached to the two nitrogen atoms of the piperazine moiety and, to the imino group of each terminal amidino function. The three equivalent guanidinium C - N bonds vary from 1.31(1) to 1.34(2) Å which are in agreement with those found in N,N'-(octane-1,8-diyl)bis(guanidinium) tetrachloroplatinate(II), the so-called $[OBG]^{2+}[PtCl_4]^{2-}$ [ from 1.295(9) to 1.314(8) Å ] [ 15 ]. The atoms C(11), N(12), N(13) and N(14) are coplanar and the propyl carbon C(10) is 0.14 Å out of this plane, to which the N(14) - C(10) is inclined at an angle of 5.40°. The diprotonated piperazinium moiety exhibits a chair conformation, with the N(1) and its centrosymmetrically related atom being 0.70 Å out of the C(2) - C(3) - $C(2^{i})$ - $C(3^{i})$ equatorial plane ( symmetry code : i = 2 - x, 1 - y, 1 - z ). The torsion angles in the N(14) - C(10) - C(20)- C(30) - N(1) sequence are in the range of 65.2 to -173.4°.

The crystal packing is characterized by four intermolecular bonds involving hydrogen atoms attached to N(12), N(13) and N(14) from the guanidinium moiety with $Cl(2^{ii})$, $Cl(3^{ii})$, $Cl(3^{iii})$ and $Cl(4^{ii})$ and another one from the hydrogen atom attached to the piperidinium N(1) atom with $Cl(1^{ii})$ ( symmetry code ii : 1 - x, - y, - z ; iii : x, - y + 1/2, z - 1/2 ).

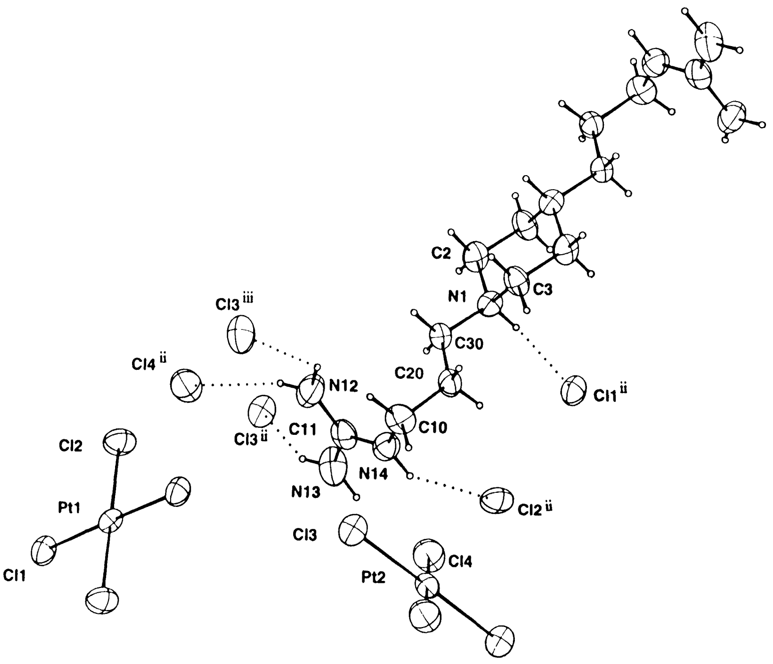

Figure 1b : Molecular structure of N,N'-bis(3-guanidinopropyl)piperazinium tetrachloroplatinate(II).

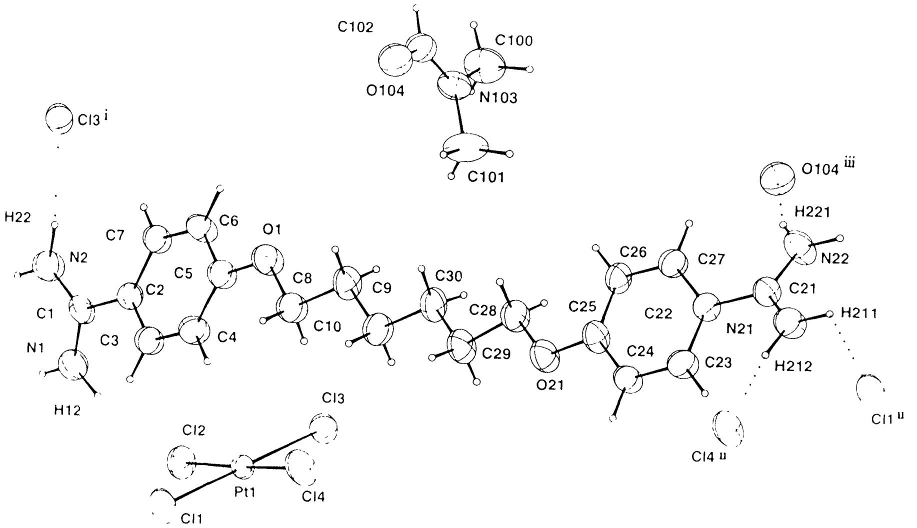

Figure 1c : Molecular structure of hexamidinium tetrachloroplatinate(II).

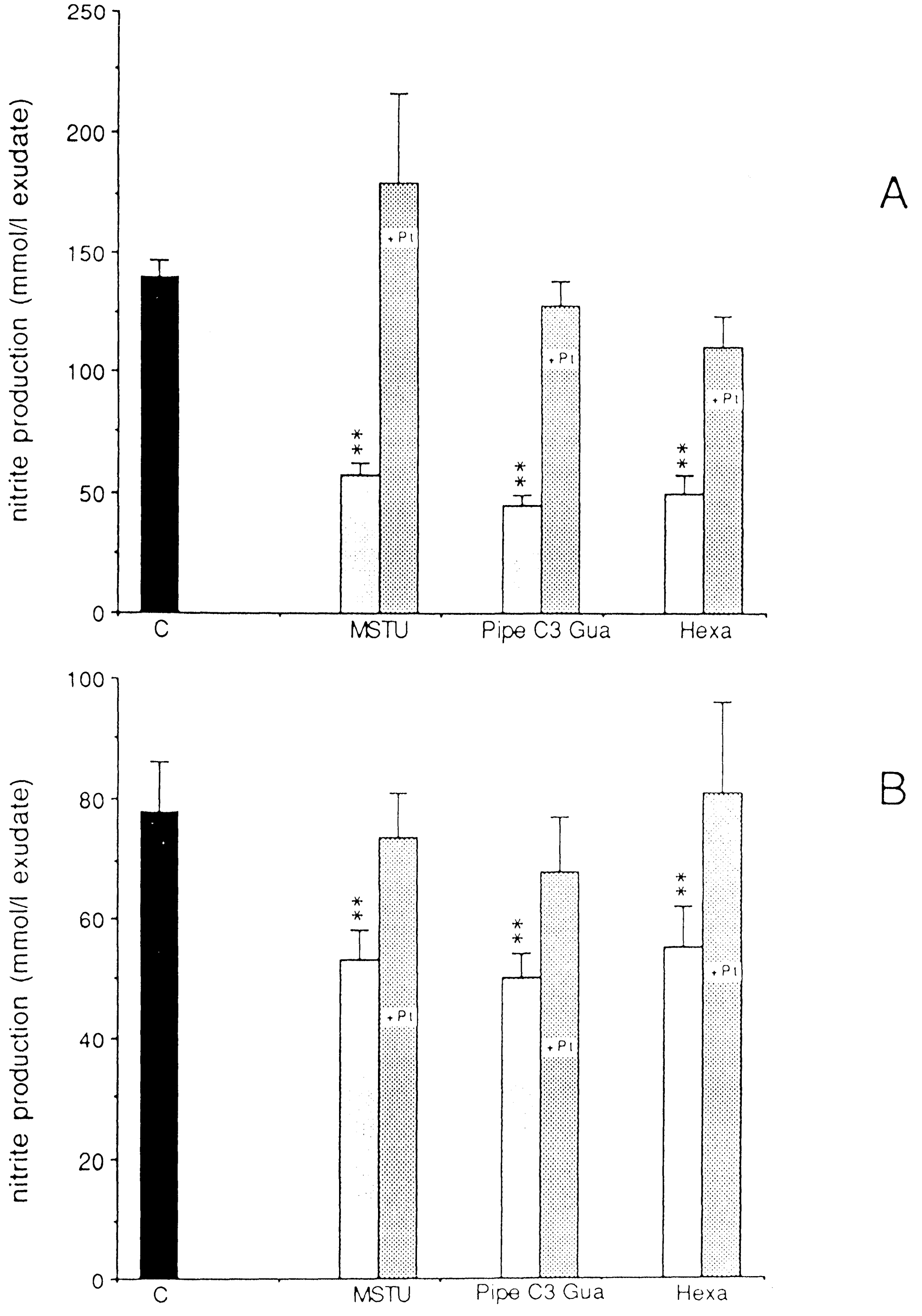

Figure 2 : Nitrite production ( m ± S.E.M. ) in the rat pleural cavity 0.5 hour ( A ) and 7 hours ( B ) after injection of 0.1 mL of carrageenan ( 10 g/L ) with or without ( C : controls ) simultaneous addition of MSTU ( S-methylisothiouronium sulfate ), PipeC3Gua (N,N'-bis(3-guanidinopropyl)piperazine dinitrate), Hexa (hexamidine diisethionate) and their tetrachloroplatinate(II) salts ( + Pt ) at the same dose of $5x10^{-5}$ M. Number of animals included in each group : 5 - 8 ; ** $p < 0.01$

2.1.3. *Description of the PtHexa structure.* ( Figure 1c )

The two phenyl rings of the hexamidinium are twisted by 14.4° with respect to each other, compared to 35° in the pentamidine complex formed with the dodecanucleotide d( CGCGAATTCGCG $)_2$ [ 18 ]. The torsion angles show that the central -$(CH_2)_6$- linker chain exhibits an all *trans* configuration, as in free pentamidine [ 19 ], in contrast with the pentamidine-dodecanucleotide complex in which the deviations from 180° pure staggered values are responsible for the shortening of the chain length to 9.13 Å ( compared to 9.75 Å value found in the crystal structure of free pentamidine [ 19] ). The amidinium groups are twisted out of the planes of the phenyl rings by 25.0 and 155.0° compared to 27° in free pentamidine and to 3 and -6° in the pentamidine complex. Moreover, in PtHexa, the dihedral angle between the two amidinium groups is 132.5°.

The crystal packing is characterized by intermolecular bonds involving Cl(2) via hydrogen H(12) attached to N(1), Cl($3^{i}$) via H(22) attached to N(2), Cl($1^{ii}$) and Cl($4^{ii}$) via the two hydrogen atoms attached to N(21) and finally O($104^{iii}$) from the dimethylformamide via H(221) attached to N(22) ( symmetry code i : 2 + x, 1 + y, z ; ii : - x, 1 - y, 1 - z ; iii : - 1 + x, y, z )

### 2.2. Anti NO synthase properties.

NO generation was assessed 0.5 and 7 hours after the induction of the inflammatory reaction. According to previous studies [ 20 ] this production correlated to the activation of constitutive and inducible NO synthases respectively.

The three non-platinum salts tested demonstrated a significant inhibition of NO generation during an experimental inflammatory reaction. This inhibition was observed on constitutive NOS ( in the early time of inflammation ) and on inducible NOS ( in the later time ) and may be beneficial in some pathological situations [ 21 ]. Moreover, the NO synthase antiinhibitor properties of these new tetrachloroplatinate(II) salts is described here for the first time, to our knowledge. These tetrachloroplatinate(II) salts did not, however, enhance nitrite production as shown in Figure 2 A and Figure 2 B. As NO exerts an ambivalent role on many physiological functions, control of its over-production may be of therapeutic value [ 22 ]. This property, demonstrated for an antiseptic compound such as hexamidine, may add some cytotoxic activity to its other properties. On the contrary, if this anti-NO property is unsuitable, formation of tetrachloroplatinate(II) salts deprived of action on NOS may be appropriate.

### 3. Experimental

3.1. *Synthesis of the tetrachloroplatinate(II) salts.*

$K_2PtCl_4$ ( Jansen Chimica ) was used as received.

3.1.1. Synthesis of bis(S-methylisothiouronium) tetrachloroplatinate(II).

Bis(S-methylisothiouronium) sulfate ($(MSTUH)_2SO_4$) ( Aldrich ) [0.556 g; 2 mmol] was dissolved in 1 M HCl ( 20 mL ) and a solution of $K_2PtCl_4$ [ 0.415 g;1 mmol ] in 1 M HCl ( 5 mL ) was added. The reaction mixture was heated and rotary evaporated to dryness. The crystalline red-brown solid was dissolved in 1 M HCl and recrystallized at room temperature. Crystals were washed with methanol ( 4 x 10 mL ) and dried at 90° C for 2 h to give 0.386g (75 % ) of $[MSTUH]_2[PtCl_4]$.

3.1.2. Synthesis of N,N'-bis(3-guanidinopropyl)piperazinium tetrachloroplatinate(II)

N,N'-bis(3-guanidinopropyl)piperazine (pipeC3Gua) dinitrate was synthesized as previously described [ 14 ]. This compound ( 0.410 g; 1 mmol ) was dissolved in 1 M HCl ( 20 mL) by heating at 50°C and stirring. To this solution was added $K_2PtCl_4$ ( 0.415 g; 1 mmol ) dissolved in 1 M HCl ( 5 mL ). The peach-coloured tetrachloroplatinate salt of PipeC3GuaH4 precipitated rapidly. The powder was filtered off and washed several times with water, ethanol, diethylether and dried at 90°C for 24 h. Yield : 0.530 mg ( 85% )

3.1.3. Synthesis of hexamidinium tetrachloroplatinate(II)

Hexamidine diisethionate ( 0.607g ; 1 mmol ) was dissolved in 1 M HCl ( 100 mL ) by heating at 70°C for 0.5 h and a solution of $K_2PtCl_4$ ( 0.415 g ; 1 mmol ) in 1 M HCl ( 5 mL ) was added. A flesh-coloured precipitate was obtained immediately, filtered and washed several times with water, ethanol and diethylether then dried at 90°C for 24 h. Crystals suitable for X-ray crystallography were obtained by recrystallization of the salt from hot DMF.

Table 1

| | | | |
|---|---|---|---|
| **Crystal Data** | | | |
| compound | PtMSTU | PtPipeC3Gua | PtHexa |
| formula | $2[C_2N_2H_7S]^+ [PtCl_4]^{2-}$ | $[C_{12}H_{32}N_8]^{4+} 2[PtCl_4]^{2-}$ | $[C_{20}H_{28}N_4O_2]^{2+}[PtCl_4]^{2-}$ $C_3H_7NO$ |
| fw(g) | 519.2 | 962.24 | 766.47 |
| shape | parallelepiped | parallelepiped | parallelepiped |
| colour | red-brown | peach | flesh |
| size, mm | 0.450, 0.400, 0.200 | 0.400, 0.180, 0.120 | 0.625, 0.500, 0.300 |
| crystal system | triclinic | monoclinic | triclinic |
| space group | P-1 | P $2_1$/c | P-1 |
| a, Å | 6.350(2) | 11.694(2) | 9.060(3) |
| b, Å | 7.278(3) | 9.497(3) | 12.787(5) |
| c, Å | 8.642(3) | 11.758(4) | 13.55(2) |
| α, ° | 72.48(2) | 90.0 | 107.156(7) |
| β, ° | 72.68(2) | 92.55(2) | 99.553(6) |
| γ, ° | 81.10(3) | 90.0 | 96.94(3) |
| V, Å$^3$ | 362.7(2) | 1304.6(7) | 1455.0(3) |
| Z | 1 | 2 | 2 |
| F(000) | 242.35 | 896.54 | 752.35 |
| ρ (calcd), g.cm$^{-3}$ | 2.38 | 2.45 | 1.75 |
| μ (MoKα), cm$^{-1}$ | 107.73 | 116.72 | 52.74 |
| **Data collection** | | | |
| 2θ range, deg | 4.0 < 2θ < 48 | 0 < 2θ < 56 | 4.0 < 2θ < 48 |
| range of h | 0 → 8 | 0 → 15 | 0 → 12 |
| range of k | -9 → 9 | 0 → 12 | -17 → 17 |
| range of l | -10→11 | -15 → 15 | -19 → 18 |
| Absorption correction | DIFABS | DIFABS | DIFABS |
| T min / T max | 0.95 / 1.00 | 0.94 / 1.00 | 0.99 / 1.00 |
| no. of rflns collected | 1747 | 3479 | 8939 |
| no. of unique rflns | 1747 | 3479 | 8939 |
| reflections used, | 1730( I > 3σ(I)) | 1378( I > 3σ(I)) | 6499(I>3σ(I)) |
| **Refinement** | | | |
| R / $R_W$ | 0.033 / 0.037 | 0.059 / 0.062 | 0.036 / 0.044 |
| Weighting Scheme | Chebyshev | Chebyshev | Chebyshev |
| Coefficient .Ar | 12.1, -9.85, 8.31 | 1.92, -0.259, 1.50 | 8.75, -2.79, 6.93 |
| GOF | 1.05 | 1.11 | 1.57 |
| $(\Delta/\sigma)_{max}$ | 0.016 | 0.015 | 0.0006 |
| $\Delta\rho_{min}/\Delta\rho_{max}$ ( e.Å$^3$ ) | -1.19 / 1.02 | -2.47 / 1.71 | -1.17 / 2.08 |
| Number of parameters | 71 | 229 | 356 |

3.2. *Structure determination.*

The refined cell constants and other relevant crystal data are presented in Table 1, together with details of the intensity measurements. The crystals were mounted, using glass fibers, on an ENRAF-NONIUS CAD4 diffractometer equipped with a graphite monochromator. The lattice parameters were refined using 25 reflections. The data were collected using the $\omega$-$2\theta$ scan technique and with Mo $K\alpha$ radiation ( $\lambda$ = 0.71073 Å ).

During the data collection, three intensity control reflections were monitored every two hours, showing no loss of intensity. The data were corrected for Lorentz and polarisation effects. The structure was solved by a combination of direct methods using SIR procedure [ 23 ] and heavy-atom techniques and refined by full-matrix least-squares method based on F, using CRYSTALS [ 24 ]. An empirical absorption correction with the program DIFABS [ 25 ] was used. Anisotropic displacement parameters were assigned to all non-H atoms. The hydrogen atoms were introduced in calculated idealized positions ( d(C-H) = 0.98 Å ) and their atomic coordinates were recalculated after each cycle. They were given isotropic thermal parameters 20% higher than those of the carbon to which they are attached. Least-squares refinements were performed by minimizing the function $\Sigma w(|F_o|-|F_c|)^2$, where $F_o$ and $F_c$ are the observed and calculated structure factors. The weighting scheme used in the last refinement cycles was $w = w'[1-(\Delta F/6\sigma(F_o)^2]^2$ where $w' = 1/\Sigma_1{}^n A_r T_r(x)$ with 3 coefficients $A_r$ for the Chebyshev polynomial $A_r T_r(x)$ where x was $F_c/F_c(max)$ [ 26 ]. Models reached convergence with $R = \Sigma(||F_o|-|F_c||)/\Sigma(|F_o|)$ and $Rw = [\Sigma w(|F_o|-|F_c|)^2/\Sigma w(F_o)^2]^{1/2}$, having values listed in Table 1. Criteria for a satisfactory complete analysis were ratios of rms shift to standard deviation less than 0.1 and no significant features in final difference maps. Details of data collection and refinement are given in Table 1. Calculations were performed with a PC CRYSTALS package program. The drawings of the molecules were generated using CAMERON [ 27 ]. The atomic scattering factors were taken from International Tables for X-ray Crystallography [ 28 ].

3.3. *Biological assays.*

3.3.1. Material.

Carrageenan $\lambda$ was obtained from Pierrefitte Auby ( Neuilly/Seine, France ). Hanks' solution ( without phenol red ), N-(1-naphthyl)ethylenediamine, and sulfanilamide were purchased from Sigma Chemical Co. ( St. Louis, Mo, USA ). Phosphoric acid was purchased from Merck ( Darmstadt, Germany ) and bis(S-methylisothiouronium)sulfate was purchased from Aldrich ( Steinheim, Germany ).

3.3.2. Animals and treatments

Male Sprague-Dawley rats weighing 180-200 g ( Dépré, Saint-Doulchard, France ) were used for all experiments [ 29 ]. Protocols were submitted to and approved by the local ethics committee.

Pleurisy was induced by intrapleural injection of 0.1 mL of 10 g/L carrageenan $\lambda$ suspension in saline [ 30 ].

Bis(S-methylisothiouronium) sulfate, PipeC3Gua dinitrate and hexamidinium diisethionate on the one hand and the corresponding tetrachloroplatinate(II) salts on the other hand were administered ( 0.1 mL ) with the inflammatory [ 14 ]. Animals were euthanized with ether, and the right pleural cavity was opened for pleural exudate collection after 30 minutes or seven hours. Samples were centrifuged at 500 g for 5 min, and the supernatants were then centrifuged at 1000 g for 20 min in order to remove exudate fibrin. Samples were then adjusted to a final volume of 1 ml with Hank's solution and stored at - 80°C until nitrite determination was carried out.

3.3.3. Nitrite determination in pleural exudate.

Nitrite was measured in pleural exudate as a parameter of NO formation [ 31 ]. Aliquots of 0.1 mL were incubated in individual wells of a 96-well plate, with 0.1 mL of Griess reagent ( 0.5 g/L N-(1-naphthyl)ethylenediamine and 5 g/L sulfanilamide in phosphoric acid ( 50 g/L )), at room temperature for 10 min. The absorbance was measured at 550 nm using a microplate reader

( Dynatech, MRX ), in comparison with the incubating medium ( Hank's solution ) without cells. Sodium nitrite was used to establish a nitrite standard curve.

3.3.4. Statistical analysis

The Mann-Whitney U test ( in the computer program Statview II ) was carried out in each group between treated and non treated rats. Results were given as a mean ± S.E.M. Differences with $p < 0.05$ were considered as significant.

Table 2 : Interatomic distances and bond angles ( Å, deg.). E.s.d's in parentheses refer to the last significant digit.

**PtMSTU**

| | | | |
|---|---|---|---|
| PT(1) - CL(1) | 2.297(1) | CL(2) - PT(1) - CL(1) | 90.01(5) |
| PT(1) - CL(2) | 2.305(1) | N(1) - C(1) - N(2) | 121.6(6) |
| N(1) - C(1) | 1.311(8) | N(1) - C(1) - S(1) | 115.4(5) |
| C(1) - N(2) | 1.302(8) | N(2) - C(1) - S(1) | 123.0(5) |
| C(1) - S(1) | 1.730(6) | C(1) - S(1) - C(2) | 103.8(3) |
| S(1) - C(2) | 1.782(7) | | |

**PtPipeC3Gua**

| | | | |
|---|---|---|---|
| PT(1) - CL(1) | 2.297(3) | CL(1) - PT(1) - CL(2) | 91.1(1) |
| PT(1) - CL(2) | 2.298(3) | CL(3) - PT(2) - CL(4) | 91.2(1) |
| PT(2) - CL(3) | 2.299(3) | C(2) - N(1) - C(3) | 109.2(8) |
| PT(2) - CL(4) | 2.290(4) | C(2) - N(1) - C(30) | 110.0(8) |
| | | C(3) - N(1) - C(30) | 113.1(8) |
| N(1) - C(2) | 1.52(1) | C(10) - N(14) - C(11) | 126.6(11) |
| N(1) - C(3) | 1.50(1) | N(1) - C(2) - C(3$^{i}$) | 110.5(9) |
| N(1) - C(30) | 1.51(1) | N(1) - C(3) - C(2$^{i}$) | 110.8(10) |
| N(12) - C(11) | 1.34(2) | N(14) - C(10) - C(20) | 111.8(9) |
| N(13) - C(11) | 1.31(1) | N(12) - C(11) - N(13) | 118.9(13) |
| N(14) - C(10) | 1.46(1) | N(12) - C(11) - N(14) | 121.5(12) |
| N(14) - C(11) | 1.33(2) | N(13) - C(11) - N(14) | 119.7(12) |
| C(2) - C(3$^{i}$) | 1.48(2) | C(10) - C(20) - C(30) | 111.8(9) |
| C(10) - C(20) | 1.51(1) | N(1) - C(30) - C(20) | 114.1(9) |
| C(20) - C(30) | 1.48(1) | | |

symmetry code : i : 2 - x, 1 - y, 1 - z

**PtHexa**

| | | | |
|---|---|---|---|
| PT(1) - CL(1) | 2.310(1) | C(2) - C(3) | 1.389(6) |
| PT(1) - CL(2) | 2.300(1) | C(2) - C(7) | 1.393(6) |
| PT(1) - CL(3) | 2.318(1) | C(3) - C(4) | 1.388(7) |
| PT(1) - CL(4) | 2.297(1) | C(4) - C(5) | 1.390(7) |
| | | C(5) - C(6) | 1.398(6) |
| O(1) - C(5) | 1.357(5) | C(6) - C(7) | 1.374(6) |
| O(1) - C(8) | 1.448(6) | C(8) - C(9) | 1.499(7) |
| O(21) - C(25) | 1.351(5) | C(9) - C(10) | 1.523(7) |
| O(21) - C(28) | 1.424(6) | C(10) - C(30) | 1.522(7) |
| O(104)- C(102) | 1.234(6) | C(21) - C(22) | 1.472(6) |
| N(1) - C(1) | 1.304(6) | C(22) - C(23) | 1.390(6) |
| N(2) - C(1) | 1.310(6) | C(22) - C(27) | 1.391(6) |
| N(21) - C(21) | 1.314(6) | C(23) - C(24) | 1.381(6) |
| N(22) - C(21) | 1.307(6) | C(24) - C(25) | 1.388(6) |
| N(103)- C(100) | 1.465(7) | C(25) - C(26) | 1.397(7) |
| N(103)- C(101) | 1.446(7) | C(26) - C(27) | 1.376(6) |
| N(103)- C(102) | 1.316(7) | C(28) - C(29) | 1.498(7) |

| | | | |
|---|---|---|---|
| C(1) - C(2) | 1.471(6) | C(29) - C(30) | 1.525(7) |

| | | | |
|---|---|---|---|
| CL(2) - PT(1) - CL(1) | 89.58(4) | C(5) - C(6) - C(7) | 119.9(4) |
| CL(3) - PT(1) - CL(1) | 178.97(4) | C(2) - C(7) - C(6) | 120.7(4) |
| CL(4 ) - PT(1) - CL(1) | 90.34(5) | O(1) - C(8) - C(9) | 107.1(4) |
| CL(2) - PT(1) - CL(3) | 91.16(4) | C(8) - C(9) - C(10) | 111.6(5) |
| CL(2) - PT(1) - CL(4) | 177.97(5) | C(9) - C(10) - C(30) | 112.1(5) |
| CL(3) - PT(1) - CL(4) | 88.95(4) | N(21) - C(21) - N(22) | 120.1(4) |
| C(5) - O(1) - C(8) | 117.6(4) | N(21) - C(21) - C(22) | 120.1(4) |
| C(25) - O(21) - C(28) | 117.8(4) | N(22) - C(21) - C(22) | 119.8(4) |
| C(100)- N(103)- C(101) | 118.1(5) | C(21) - C(22) - C(23) | 121.1(4) |
| C(100)- N(103)- C(102) | 120.6(5) | C(21) - C(22) - C(27) | 120.3(4) |
| C(101)- N(103)- C(102) | 121.3(5) | C(23) - C(22) - C(27) | 118.6(4) |
| N(1) - C(1) - N(2) | 120.0(4) | C(22) - C(23) - C(24) | 120.6(4) |
| N(1) - C(1) - C(2) | 119.4(4) | C(23) - C(24) - C(25) | 120.3(4) |
| N(2) - C(1) - C(2) | 120.7(4) | O(21) - C(25) - C(24) | 116.5(4) |
| C(1) - C(2) - C(3) | 119.0(4) | O(21) - C(25) - C(26) | 124.1(4) |
| C(1) - C(2) - C(7) | 121.8(4) | C(24) - C(25) - C(26) | 119.4(4) |
| C(3) - C(2) - C(7) | 119.2(4) | C(25) - C(26) - C(27) | 119.7(4) |
| C(2) - C(3) - C(4) | 120.8(4) | C(22) - C(27) - C(26) | 121.2(4) |
| C(3) - C(4) - C(5) | 119.4(4) | O(21) - C(28) - C(29) | 109.9(4) |
| O(1) - C(5) - C(4) | 123.9(4) | C(28) - C(29) - C(30) | 109.6(5) |
| O(1) - C(5) - C(6) | 116.1(4) | C(10) - C(30) - C(29) | 115.0(5) |
| C(4) - C(5) - C(6) | 120.0(4) | | |

Other crystallographic details, comprising fractional atomic coordinates, hydrogen bonds, thermal parameters, and tables of observed and calculated structure factors are available on request.

Acknowledgments.

The authors would like to thank Mrs M. Lenoir and Mr O. Muntaner for their excellent technical assistance.

We are grateful to Chauvin Laboratory ( France ) for its generous gift of hexamidine diisethionate.

**REFERENCES**

1 R. M. Palmer, A. G. Ferrige, S. Moncada, *Nature*, **327** (1987) 524

2 L. J. A. Ignarro, *Rev. Pharmacol. Toxicol.*, **30** (1990) 535

3 S. Moncada, A. Higgs, *New Engl. J. Med.*, **329** (1993) 2002

4 S. R. Vincent, H. Kimura, *Neuroscience*, **4** (1992) 755

5 S.J. Green, C. A. Nancy, *Curr. Opin. Infect. Dis.*, **6** (1993) 384

6 J. Zhang, V. Dawson, T. Dawson, S. Snyder, *Science*, **263** (1994) 687

7 R. G. Kilbourn, S. S. Gross, A. Jubran, J. Adams, D. W. Griffith, R. Levi, R.F. Lodato, *Proc. Natl. Acad. Sci. USA*, **87** (1990) 3629

8 H. K. Zhang, W. Fast, M. A. Marjetta, P. Martasek, R. B. Silverman, *J. Med. Chem.*, **40** (1997) 3869

9 E. P. Garvey, J. A. Oplinger, E. S. Furfine, P. J. Kiff, F. Laszlo, B. J.R. Whittle, R. G. Knowles, *J. Biol. Chem.*, **272** (1997) 4959

10 G. J. Southan, C. Szabo, C. Thiemerman, *Br. J. Pharmacol.*, **114** (1995) 510

11 D. J. Wolff, D. S. Gauld, M. J. Neulander, G. J. Southan, *J. Pharmacol. Exp. Ther.*, **288** (1997) 265

12 C. Szabo, G. J. Southan, C. Thiemermann, *Proc. Natl. Acad. Sci. USA*, **91** (1994) 12472

13 G. J. Southan, D. Gauld, A. Lubeskie, B. Zingarelli, S. Cuzzocrea, A. L. Salzman, C. Szabo, D. J. Wolf, *Biochem. Pharmacol.*, **54** (1997) 406

14 M. Roch-Arveiller, C. Regnault, J. P. Giroud, G. Morgant, J. C. Lancelot, C. Saturnino, D. Perrine, Nguyen-Huy Dung, *Eur. J. Clin. Chem. Clin. Biochem.*, **35** (1997) 743

15 B. Viossat, Nguyen-Huy Dung, X. Labouze, G. Morgant, J. C. Lancelot, D. Perrine, M. Robba, *J. Inorg. Biochem.*, **65** (1997) 163

16 C. H. Stam, *Acta Cryst.*, **15** (1962) 317

17 O. Kennard, J. Walker, *J. Chem. Soc.*, (1963) 5513

18 K. J. Edwards, T. C. Jenkins, S. Neidle, *Biochemistry,* **31** (1992) 7104

19 P. R. Loewe, C. E. Sansom, C. H. Schwalbe, M. F. G. Stevens, *J. Chem. Soc., Chem. Commun.*, (1989) 1164

20 C. Regnault, M. Roch-Arveiller, I. Florentin, J. P. Giroud, E. Postaire, M. Delaforge, *Inflammation*, **20** (1996) 613.

21 J. Pfeilschifter, W. Eberhardt, R. Hummel, D. Kunz, H. Mühl, D. Nitsch, C. Plüss, G. Walker, *Cell Biol. International*, **20** (1996) 51

22 R. Kilbourn, *Molec. Med. Today*, **2** (1996) 324

23 M. C. Burla, M. Camalli, G. Cascarano, C. Giacovazzo, G. Polidori, R. Spagna and D. Viterbo, *J. Appl. Cryst.*, **22** (1989) 389

24 D. J. Watkin, J. R. Carruthers and P. W. Betteridge, *CRYSTALS- User Guide. Chemical Crystallography Laboratory*, Univ. of Oxford, England, 1985.

25 N. Walker and D. Stuart, *Acta Crystallogr.* **A39** (1983) 158

26 E. Prince, *Mathematical Techniques in Crystallography*, Springer-Verlag, Berlin, (1982)

27 L. J. Pearce, D. J. Watkin, C. K. Prout,. *CAMERON. A program for plotting molecular structures. Chemical Crystallography Laboratory*, 1992, Univ. of Oxford, England.

28 International Tables for X-ray Crystallography, Vol IV, pp. 99-101 (1974)

29 J. P. Giroud, M. Roch-Arveiller, O. Muntaner, *Nelle Rev. Fse. Hematol.* **20** (1978) 535

30 M. Roch-Arveiller, C. J. Dunn, J. P. Giroud, *J. Pharmacol.* 8 (1977) 461

31 M. A. Marletta, P. S. Yoon, R. Iyengar, C. D. Leaf, J. S. Wishnok, *Biochemistry,* **27** (1988) 8706

**Received: March 12, 1998 - Accepted: March 25, 1998**

